# Electrically Controlled Enrichment of Analyte for Ultrasensitive SERS-Based Plasmonic Sensors

**DOI:** 10.3390/nano12050844

**Published:** 2022-03-02

**Authors:** Georgii Pavliuk, Alexey Zhizhchenko, Oleg Vitrik

**Affiliations:** 1 Far Eastern Federal University, 8 Sukhanova St., 690950 Vladivostok, Russia; oleg_vitrik@mail.ru; 2 Institute for Automation and Control Processes, 5 Radio St., 690041 Vladivostok, Russia; g89leksig@mail.ru

**Keywords:** superhydrophobic surface, analyte enrichment, electrostatic action, plasmonic sensors, SERS

## Abstract

Recently, sensors using surface-enhanced Raman scattering (SERS) detectors combined with superhydrophobic/superhydrophilic analyte concentration systems showed the ability to reach detection limits below the femto-molar level. However, a further increase in the sensitivity of these sensors is limited by the impossibility of the concentration systems to deposit the analyte on an area of less than 0.01 mm^2^. This article proposes a fundamentally new approach to the analyte enrichment, based on the effect of non-uniform electrostatic field on the evaporating droplet. This approach, combined with the optimized geometry of a superhydrophobic/superhydrophilic concentration system allows more than a six-fold reduction of the deposition area. Potentially, this makes it possible to improve the detection limit of the plasmonic sensors by the same factor, bringing it down to the attomolar level.

## 1. Introduction

Various fields of microbiology, biochemistry and medicine require the development of techniques that allow us to carry out express detection substances in their aqueous solutions at an ultra-low concentration [1,2,3,4,5,6,7]. Significant attention in these studies is paid to plasmonic sensors [4,5,6,7,8,9,10,11,12,13], mainly due to their non-invasiveness and ultra-high sensitivity [11,14,15,16,17,18]. Operation of these types of sensors is based on the phenomenon enhancement of Raman response from analyte molecules near “hot spots” at special nanostructures [19,20,21]. However, such “hot spots” do not cover the entire SERS-active area of the sensor. As a result, the analyte molecules are not always located near the enhanced regions. Moreover, at ultra-low concentration, the analyte in solution is highly dispersed throughout the volume. This may cause low probability of its hit into a “hot spot” per unit time [22,23]. In order to accumulate sufficient signal, a long wait is required. Performance plasmonic sensors may be significantly increased by a targeted deposition of analyte molecules on the surface of a sensitive element.

One of the effective ways to achieve this is to use superhydrophobic surfaces (SHPS) [24]. Due to the low adhesion of SHPS, the contact line of a droplet of aqueous solution slides over the surface during evaporation. As a result, the analyte is localized in an extremely small area, thereby providing a high deposition density [22,24,25,26,27,28,29,30]. To protect the droplet from rolling, as well as to localize the deposition area at a definite point at the SHPS, the latter is manufactured with a non-uniform distribution of surface energy. The highest efficiency is demonstrated by structures in which the surface energy gradient is achieved at the expense of a special hybrid design combining superhydrophilic and superhydrophobic surface properties [27,28,31]. In this case, a peripheral part is formed on the substrate, consisting of superhydrophobic micropillars, as well as a central part, in the form of a superhydrophilic target of a small area. The latter in these structures is an attractor that holds a droplet during the entire evaporation time, as a result of all the analyte being transported and deposited on the target surface, where previously [27] during deposition [15] set the properties of SERS activity. An important advantage of this type structure is the simplicity of their manufacture through laser processing of the workpiece surfaces, without resorting to expensive and resource-intensive methods. Despite the simplicity of manufacture, such structures already demonstrated their ability to detect SERS responses from analytes in solutions with femto- [31] and even atto-molar concentration [15].

The efficiency of hybrid superhydrophobic/superhydrophilic structures in detecting analytes at ultra-low concentrations is determined, primarily, by the area of the target: the smaller the area, the denser and more uniform is the distribution of the molecules studied on its surface. A further increase in the detection efficiency may be achieved by improving the SERS sensitivity. Several studies are devoted to methods of increasing the latter [32,33,34,35,36]. However, according to the well-aimed remark, “*even the most optimally designed SERS platforms are essentially nullified if the target analytes cannot access the electromagnetic hotspots*” [21] we choose to focus on the first aspect: to achieve the smallest target area in this work.

Despite the fact that specific implementations of hybrid superhydrophobic/superhydrophilic structures with a central target may vary considerably in the materials used and in the details of the surface design, they all currently demonstrate a similar limit for minimizing the analyte deposition area, which is about 0.01 mm^2^ [15,27,32].

We demonstrate more than a six-fold reduction in the deposition area achieved by a non-uniform electrostatic field on evaporating an electrically neutral droplet. Geometry modification of the hybrid superhydrophobic/superhydrophilic structures in the case of the electrostatic action is also demonstrated.

## 2. Methodology

**Figure 1:**
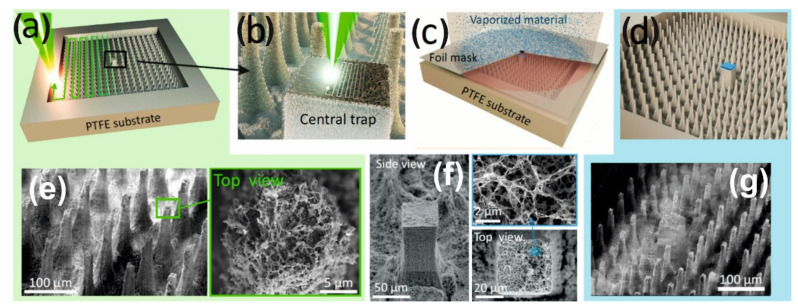
Manufacturing process of the initial design LITHS/S- structures. (**a**) Manufacturing of the peripheral superhydrophobic pillars by laser pulses of 0.3 μJ; (**b**) manufacturing of nano-rough relief of the central target by laser pulses of 0.1 μJ; (**c**) coating the target in layers Ti, Au, and SiO_2_ by electron beam sputtering through a mask; (**d**) manufactured LITHS/S- structure; (**e**–**g**) SEM images of superhydrophobic pillars, central target and LITHS/S- structure respectively.

To create the superhydrophobic peripheral part of the hybrid structures we use a single piece substrate of polytetrafluoroethylene (PTFE) on the surface of which superhydrophobic pillars are made by direct femtosecond (fs)-laser printing [27,37,38,39,40] (Figure 1a). Moreover, the surface of these pillars in the process of laser ablation additionally acquires a multimodal roughness [28]. As a result, the value of the contact angle at such peripheral part is very large—about θ~164°. The central target is also formed due to (fs)-laser printing (Figure 1b) in the form of a larger pillar with a nano-rough surface, which is then covered (Figure 1c) with thin layers of Ti (50 nm), Au (150 nm) thickness, and SiO_2_ (10 nm) (Figure 1d) to make its surface superhydrophilic. (The superhydrophilic properties can be achieved with a significantly thinner layer of SiO_2_ (2–5 nm), however, we used a thicker layer to avoid quenching the luminescence of the R6G analyte to clearly demonstrate the effect of enrichment).

**Figure 2:**
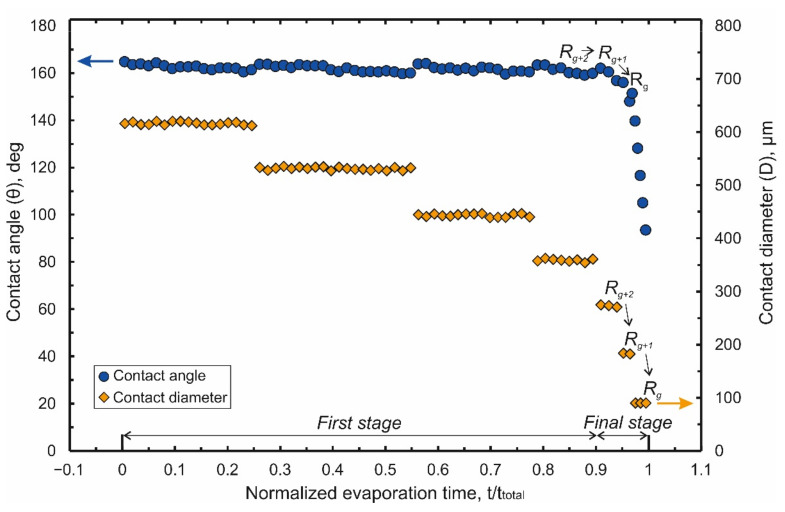
Dependences of the contact angle and the contact diameter of a droplet on the normalized evaporation time in the initial design LITHS\S-structure in the absence of an electric field.

The process of droplet evaporation on this original design of laser-induced Teflon hybrid superhydrophobic\superhydrophilic-structure (LITHS\S-structure) may be analyzed in the absence of an electrostatic field. Results of the measurements of the droplet contact angle (CA) θ and contact diameter (CD) D versus normalized evaporation time τ are shown in Figure 2. (Normalized evaporation time means ratio of the current time t to the total evaporation time t_total_ : τ = t/t_total_).

As shown in Figure 2, the process of droplet evaporation can be conditionally divided into two stages. During first stage (τ < 0.9) a gradual decrease in the contact diameter (Figure 2) is observed. The CD decrease has stepwise nature with value of decrement being a multiple the spatial period of pillars (Λ). At the same time, the contact angle oscillates near an initial value (θ~164°). It slightly decreases during the subinterval when the contact diameter remains almost constant and each time returning to the initial value at the moment leap of the CD. This behavior of the droplet is explained by a change in the balance of pinning forces $F_{P}$ (the force of droplet fixation to superhydrophobic pillars. It may be viewed as an analogue of the friction force during droplet evaporation) and depinning forces $F_{\mathrm{dp}}$ (the force arising due to surface tension and tending to pull the droplet to the center of the contact region). For a fixed CD, a decrease in the initial volume $V_{0}$ of an evaporating droplet leads to a decrease in the contact angle, resulting in an increase of the depinning force according to:

(1) $$F_{\mathrm{dp}}\;\sim \;\sigma \;\left(\cos\;\theta -{\;\cos\;\theta }_{Y}\right),$$

where $\theta_{Y}$ is an equilibrium contact angle, σ is a vapor-liquid surface tension. When CA decreases to a value of the receding angle $\theta_{r}$ [41,42] the condition $|F_{\mathrm{dp}}|>\left|F_{P}\right|$ is true. As a result, the contact line (CL) recedes, stopping on the next set of pillars closer to the central target. During almost the entire first stage, the central target remains inside the contact region. Sometimes part of the contact line is fixed on the target, and part on remote pillars, located at a distance of several Λ.

**Figure 3:**
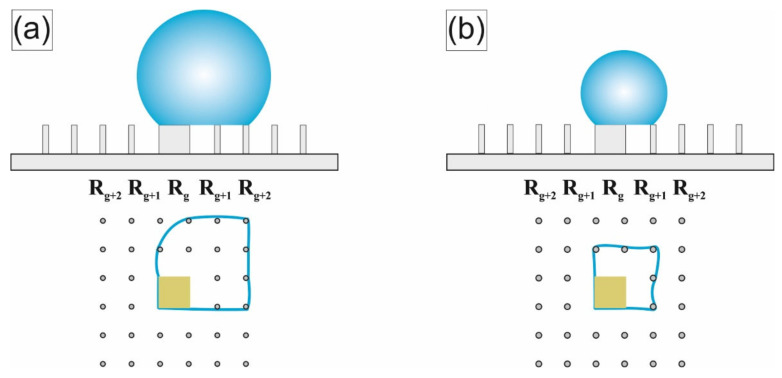
Changes in the position of the contact line at the final stage of evaporation. (**a**) CL in position R_g+2_, (**b**) CL in position R_g+1_.

The final stage (τ = 0.9 ÷ 1) is defined by the moment when the contact line is fixed partially on the target and partially on the penultimate row of pillars near it (hereinafter we will denote this row as R_g+2_) in Figure 3a. In Figure 2, this stage is characterized by a noticeable decrease in the time intervals between leaps of CD and CA. The influence of the target on the evaporation process becomes more significant in this stage. Depending on the target perimeter (~4 g), which sets the strength of the droplet attachment to it, two different scenarios are observed.

An unsuccessful scenario is observed when the droplet is faintly attached to the target, at g < 90 μm. In this case, at the final stage, the transition to the Wenzel state begins [43]. As a result, two (or even several) centers of attachment for the droplet are formed: the target itself and the contact regions in the Wenzel state. Therefore, at the end of evaporation, the contact line either breaks off completely from the target and the precipitate falls outside the target (at g < 70 μm), or the droplet stretches between the centers of attachment (or even breaks apart) and the deposition region is partly located on the target and partly on the superhydrophobic pillars adjacent (70 µm < g < 90 µm).

For successful scenario (at g ≥ 90 μm), the force of the droplet fixing to the target significantly exceeds the force of its fixation on the pillars. Accordingly, the contact line is redistributed first onto the last row of pillars in front of the target (denoted further, as R_g+1_, Figure 3b) at the moment τ_2→_ ≈ 0.94. At moment τ_1→_ ≈ 0.97 CL reaches the target surface, on which during the remaining time (τ = 0.97 ÷ 1) all the dissolved analyte is deposited. Thus, the area limit of the analyte deposition region (S_d_) equals to g^2^ = 0.0081 mm^2^. This area is just slightly smaller than that previously achieved for other hybrid superhydrophobic/superhydrophilic structures with a central target [15,31]. Therefore, we will further discuss measures to reduce this area.

Although the area of deposition $S_{d}$ is an important parameter characterizing the ability of the structure to concentrate the analyte, nevertheless, the final deposition density depends not only on $S_{d}$ value, but also on the initial volume of the droplet $V_{0}$. Therefore, in our previous works we use a more universal parameter—the concentration factor [27,28].

(2) $$K_{c}=\frac{V_{0}}{S_{d}}.$$

The larger this factor, the higher the final density of deposition on the target at the same initial concentration of the analyte. For the LITHS/S-structure under consideration and other structures of this type [28], the concentration factor reaches $K_{c}\;\approx \;600\;\mathrm{mm}$.

From Equation (2), it follows that an increase in the concentration factor could be achieved by increasing the initial volume of the droplet. However, if initial volume is too large the evaporation time of the droplet is also greatly increased. Meanwhile, according to references [44,45,46], the Cassie-Baxter state [47] is metastable. For the LITHS/S-structures under consideration, a droplet of an initial volume larger than 5 μL (t_total_ > 3000 s) has sufficient time to pass to the Wenzel state [43] before the process of its evaporation is completed. In the Wenzel state, the motion of the contact line stops. This leads to a significant increase in the diameter of the analyte deposition region to ~200 μm, which compensates for the positive effect. Therefore, we achieve to increase of the concentration factor by reducing the transverse dimensions of the final deposition region.

**Figure 4:**
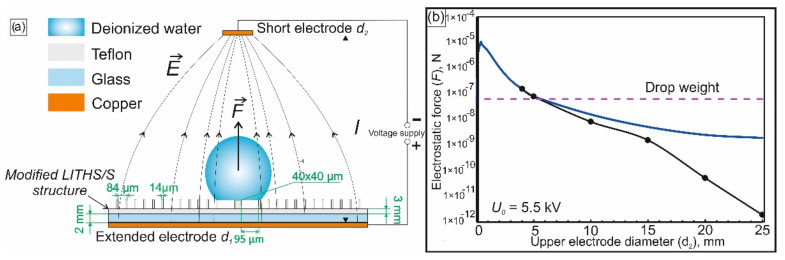
(**a**) System for droplet evaporation in a non-uniform electrostatic field (for clarity, the scale is not observed). (**b**) illustrates the dependence of the electrostatic force acting on the droplet at the time of the R_g+2_ → R_g_ transition on the diameter of the upper electrode. Solid curve—analytical results, numerical simulation data are shown by dots.

The balance of the droplet fixation forces of the target and of the pillars adjacent to it at the final stage of evaporation is now discussed. The position of the droplet at the moment when the CL is partially attached to the target and partially attached to the penultimate row of pillars (denoted further, as R_g+2_) is schematically shown in (Figure 3a). In the course of evaporation, at time τ_2→_ the contact angle approaches the critical (receding) value. As a result, the depinning force increases, which leads to the droplet moving to the last row of pillars in front of the target, (Figure 3b). After the droplet reaches the last row, the total perimeter of the droplet’s contact with the pillars is significantly reduced and becomes commensurate with the perimeter of the target. Therefore, the influence of the central target on the evaporation process is significantly enhanced. The transition R_g+2_ → R_g+1_ (as can be seen from (Figure 2) is accompanied by a jump in the CA to an initial value that is approximately equal to the equilibrium value of CA. As a result, the depinning force immediately after this transition (according to Equation (1)) decreases to almost zero. Although the contact angle in the process of further droplet evaporation can decrease again; nevertheless, for simplicity, we assume that at this stage the effect of the depinning force on the droplet placement to the target may be neglected. The displacement mainly depends on the balance between the forces of the droplet fixation on the target ($F_{p}^{\mathrm{target}}$) and nearby pillars ($F_{p}^{\mathrm{pillars}}$). These forces are proportional to the perimeter of the relief details with which the droplet is in contact [41,42]. Therefore, $F_{p}^{\mathrm{target}}$ is proportional to the target perimeter, and the $F_{p}^{\mathrm{pillars}}$ to the perimeter of the contact with the pillars of the R_g+1_ row. Excessive reduction of the target perimeter leads to a weakening of the force $F_{p}^{\mathrm{target}}$ and the realization of the unsuccessful scenario. The unsuccessful scenario can be avoided if, simultaneously with a decrease in the target perimeter, the perimeter of contact between the droplet and the pillars nearest to it is also reduced, for example, by reducing the diameter of the latter. However, if the diameter of the pillars is too small, their capillary force is greatly reduced and the probability of the “impalement” effect increases. That is, the droplet being pushed onto the pillar with a subsequent transition to the Wenzel state [48]. Besides, very thin pillars have low mechanical strength and may be damaged under the action of pinning/depinning forces. Finally, reducing the diameter of the relief features on the LITHS/S structure will require unnecessary efforts to improve the resolution of the laser technology used for the LITHS/S structure manufacturing. Therefore, we propose a different solution, which consists of modifying the design of the original LITHS/S-structures by removing a row of pillars R_g+1_, adjacent to the target (Figure 4a). Removal of this adjacent row enables thereby removing their pinning effect on the droplet. It is assumed that the powerful pinning force of the central target allows to displace the contact line off the pillars of the R_g+2_ row in the direction of the central target at time τ_2→_. However, due to the decrease in the capillary force for the area devoid of pillars, the contact line of the droplet cannot retreat to the required distance. Additional experiments show that the droplet inevitably falls into the created local cavity. This position corresponds to the Wenzel–Cassie state [49,50], in which a further decrease in CD becomes impossible. As a result, the diameter of the final deposition area turns out to be ~2Λ ≈ 170 μm, that is, unacceptably large.

We assume that a droplet falling into the cavity formed on the modified LITHS/S-structure can be avoided when there is an additional vertical force that acts on the droplet, which can compensate for the lack of capillary force. This additional force may facilitate the transfer of the entire droplet from a row of R_g+2_ pillars directly to the central target (R_g+2_ → R_g_ transition). To create such a force, we propose to place an electrically neutral evaporating droplet in a non-uniform electrostatic field (NEF). Due to the polarization of water in the electric field, the drop acquires a dipole moment and subsequently this dipole begins to “pull up” into the region with a higher NEF gradient. The region with a higher NEF gradient is located so that the droplet tends upward, to compensate for the lack of capillary force.

In this work, the NEF is created by applying a potential difference to the upper and lower disc Cu electrodes that are significantly different in diameter (Figure 4a). In this case, the droplet and the substrate are isolated from the electrodes. The droplet is insulated by an air gap, and the PTFE surface is insulated with a polytetrafluoroethylene layer and an additional glass plate (the latter is also needed in order to shift the droplet center slightly upward to the region with a slightly higher NEF gradient). The insulation is needed in order to avoid changing the wetting PTFE surface under the action of the charges flowing into the contact area of the droplet and substrate [51,52]. This electrowetting effect, as discussed below, may arrest the process of the evaporative reduction of the contact region, which is undesirable for the problems being solved.

If the electric field gradient is constant or changes little within the droplet size, the force of electrostatic action on the droplet can be calculated as:

(3) $$F_{el}=\pi \frac{D_{\mathrm{water}}^{3}}{8}\varepsilon_{\mathrm{air}}\frac{\varepsilon_{\mathrm{water}}-\varepsilon_{\mathrm{air}}}{\varepsilon_{\mathrm{water}}+\varepsilon_{\mathrm{air}}}\nabla E^{2},$$

where **E**—field strength; $D_{\mathrm{water}}$—droplet diameter; $\varepsilon_{\mathrm{water}}$—dielectric constant of water; $\varepsilon_{\mathrm{air}}$—dielectric constant of air; ∇—gradient operator. Analytical calculation of the NEF distribution and the corresponding electrostatic force $F_{\mathrm{el}}$ is performed using the method of images [53,54]. Within this framework, it may be assumed that a mirror image of the upper small electrode (diameter d_2_) with the opposite charge appears in the lower electrode, of an infinitely large diameter (d_1_) (Figure 4a). Using method of images, the field in the interelectrode space will be described by the well-known solution for the dipole field [54]. This field is created in this case by the upper and “mirror image” electrodes with a distance of 2l between them (where l is the gap between the upper and lower electrodes).

Analytical calculations also take into account the leaps in the vertical component of the electric field at the boundaries of air (ε = 1), polytetrafluoroethylene (ε = 2.3) and glass (ε = 8). The upper electrode is placed at a distance l = 7 mm, that at least several times larger than the initial droplet diameter (~2 mm). The diameter of the lower electrode d_1_ is chosen to be equal to 50 mm, which is significantly larger than distance l, and the diameter of the upper electrode varies between 0.01 and 25 mm. When selecting a particular value d_2_, we assume that at the time τ_2__→_ of transition R_g+2_→ R_g_ a value of the force acting on the droplet must be the same order of magnitude as its weight (P ≈ 62.3 nN). It was also taken into account that the maximum voltage value should be significantly lower than the experimentally measured breakdown voltage of the interelectrode gap (U_bd_ ≈ 10 kV). Therefore, during the simulation, a significantly lower value of U_0_ ≈ 5.5 kV was chosen as the assumed operating voltage.

To refine analytical results at sufficiently large diameters of the upper electrode when it becomes comparable to the diameter of the lower electrode (the lower electrode in this case could not even be considered “infinite”, which is not provided for by the analytical model), we resorted to numerical calculations using the COMSOL software version 5.5.

## 3. Results and Discussion

The results of the magnitude calculations of the electrostatic lifting force acting on the droplet are shown in (Figure 4b). As expected, the analytical and numerical data are in good agreement in the region of small values of d_2_ and begin to diverge where this diameter becomes comparable to the diameter of the lower electrode d_1_.

From the results presented in (Figure 4b), it can be seen that at the moment of the R_g+2_ → R_g_ transition, the electrostatic force exceeds the droplet weight when the diameter of the upper electrode is in the range of 0.02 ÷ 5 mm. If d_2_ < 0.02 mm, the NEF gradient increases significantly near the upper electrode. Near the lower one, where the droplet is actually located, the field is almost uniform. Accordingly, the lifting force acting on the droplet from the side of the NEF also becomes negligible. In the opposite situation, when the diameter d_2_ becomes more than 5 mm and continues to increase, the value of the field gradient decreases in the whole interelectrode space. At the moment when the diameter of the upper electrode increases to the size of the lower one, the gradient disappears, since in this case we obtain a uniform field of a flat capacitor. As a result, there is no electrostatic force acting on the droplet. Data on (Figure 4b) also show that, at the boundaries of the considered range, when the diameter d_2_ becomes equal to ~0.01 mm or ~5 mm, the lifting force is compared with the weight of the droplet. If the diameter d_2_ is chosen near the lower value from this range, then electric field lines will be too concentrated near the upper electrode, which can lead to a local electrical breakdown of the adjacent air layer. This leads to the instability of the force action on the drop and, often, to electrode damage. In addition, the manufacturing of an electrode with a diameter ~5 mm is much simpler. Therefore, a diameter of 5 mm was selected for the system described herein. For these geometrical parameters the system allows relatively large deviations from the selected interelectrode voltage of 5.5 kV. Unsuccessful outcome of evaporation is observed at U > 6.3 kV, when the droplet breaks off and flies upward from the modified LITHS/S-structure, or at U < 5 kV, when it falls into the cavity, between the target and a row of R_g+2_ pillars.

In addition to the geometric parameters of the electrodes and the potential difference between them, the temporal parameters of the interelectrode voltage pulse are also of great importance. Microscopic observations show that an excessive duration of NEF action on a droplet placed in the considered system leads first (after ~30 s) to the transition of the latter to a micro-Wenzel state in which it envelops micro-roughnesses on the surface of the pillars. Subsequent (after ~100 s) to a transition to a mixed Wenzel–Cassie state [45,49], the droplet begins to permeate deeper and deeper into the gaps between the pillars. After another ~200 s, the droplet completely fills these gaps, passing in the Wenzel state. In other words, the transition to the Wenzel state occurs an order of magnitude faster than in the absence of a field for drops of the same volume. We assume that this circumstance is due to the gradual accumulation of surface charge in the contact area (which, among other things, is influenced by the increased humidity of the environment near the evaporating droplet) [52].

**Figure 5:**
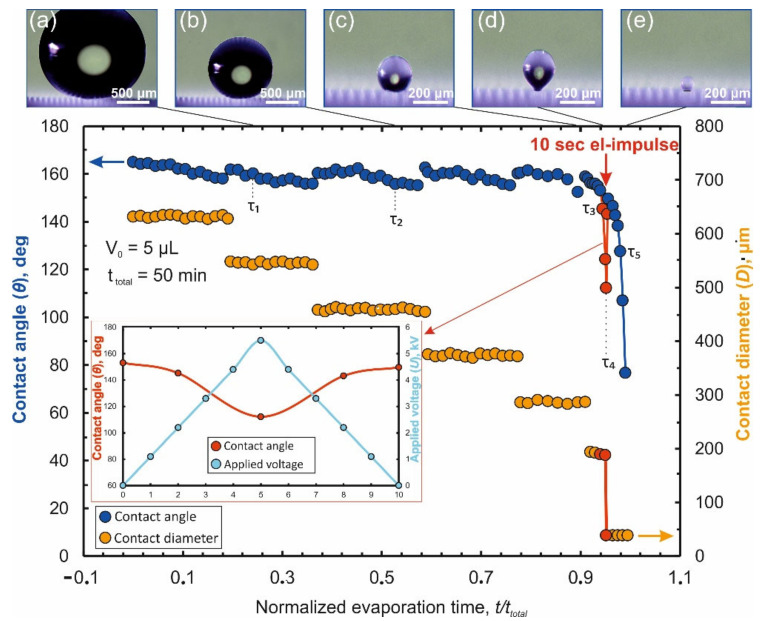
Changes in CD and CA depending on the normalized evaporation time on a modified LITHS/S-structure (g = 40 μm, Λ = 84 μm, d = 14 μm) with action a pulse of non-uniform electric field. Insets (**a**–**e**)—photomicrographs of a droplet at different evaporation moments. Inset at the bottom—change in CA under electrostatic action, the time on the axis is counted from the moment the interelectrode voltage pulse supply.

Wherein, for a short duration of stay a droplet with a Teflon substrate in an electrostatic field, for example, for no more than 10 s, the charge in the contact region does not have time to accumulate. This guarantees the stability of the Cassie wetting state, which determines the choice of the upper limit time of action NEF in our experiments. It was also found that too abrupt switching on/off of the field can lead to uncontrolled movement of the droplet over the superhydrophobic surface: rolling or even complete separation of the droplet upward from the surface. This problem is solved by gradually increasing/decreasing the voltage on the electrodes in such a way that at first, within 5 s, the voltage rises linearly to 5.5 kV and then, according to the same law, decreases to zero. As a result, full pulse duration does not exceed the selected limiting time of the action NEF (Figure 5). Experiments also confirmed the assumption that the direction of the force acting on the droplet does not depend on the polarity voltage applied to electrodes and is determined only by the direction of the NEF gradient vector.

Since the total duration of the formed pulse turns out to be significantly shorter than the total duration of the evaporation final stage (equal to ≈300 s at V_0_ = 5 μL), the choice of the pulse timing is of great importance. In order to successfully transfer the contact line to the target, it is necessary to choose the onset of the pulse in such a way that the voltage peak is reached a few seconds prior to the time of natural separation τ_2→_ of the contact line from the row of pillars R_g+2_. In this case, the evaporation of a 5 μL droplet with a dissolved Rhodamine 6G (R6G) organic dye used as an analyte at an initial concentration of 10^−10^ M illustrated in Figure 5.

**Figure 6:**
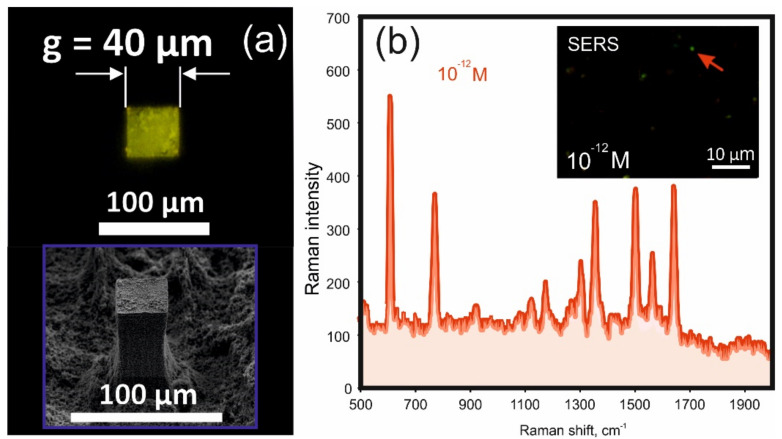
Enrichment results demonstration. (**a**) The fluorescence image of deposited 10^−10^ M R6G solutions onto the central target (top view). Inset demonstrate the scanning electron microscopy (SEM) image of the central target (side angle of 40°). (**b**) The SERS spectrum measured from Rhodamine 6G (R6G) molecules (initial analyte concentration is 10^−12^ M. The inset demonstrates the pattern of SERS responses over illumination by a pump beam with λ = 0.532 μm.

As can be seen on Figure 5, from the moment the NEF is switched on, CA decreases as the interelectrode voltage increases which, according to Equation (1), accordingly leads to an increase in the depinning force directed to the target. Microscopic observations (inset “c” in Figure 5) show that at this time the droplet is elongated (which is the direct reason for the decrease in CA) and rises upward. At the moment when the maximum voltage is reached, the droplet is detached from the pillars of the R_g+2_ row and the contact line is completely transferred to the surface of the central target (inset “d” in Figure 5). This transition is accompanied by a leap in the CA up to a value close to the initial one, but not yet equal to it, since the droplet still retains its elongated shape under the action of the field. Further, as the voltage decreases, the CA is restored to its initial value, but this happens when the droplet is already entirely on the target surface. The droplet continues to be located on the target even after the field stops acting (inset “e” in Figure 5), as a result of which all the analyte is deposited on the surface of the target, which is also illustrated by photoluminescence in Figure 6a.

This scenario is observed until the lateral dimension of the target is not lower than 40 μm. For targets with smaller dimensions the pinning force is no longer sufficient for the “successful” completion of the deposition process even under the action of NEF. As a result of which the analyte is again redistributed between the target and adjacent pillars of the superhydrophobic surface. As one can see, g = 40 μm and, accordingly, S_D_ = 0.0016 mm^2^ is the limit we have reached to minimize the analyte deposition area when using the proposed approach. This result allows an increase of the concentration factor up to $K_{c}$ ≈ 3125 mm.

As mentioned above, to prevent photoluminescence signal quenching, the central target is covered with a rather thick SiO_2_ buffer layer. This inevitably worsens the SERS response of the target, in particular due to the blocking of the chemical mechanism of enhancement of Raman scattering (although it is possible that some analyte molecules can penetrate cracks in the buffer coating, up to direct contact with the metal). We took this step to visually demonstrate the results of targeted analyte deposition on a central small-sized target, which is illustrated by the fluorescence image of the deposition of 10^−10^ M R6G (Figure 6a). However, the target surface of the LITHS/S structure exhibits pronounced SERS activity due to apparently sufficient electric field enhancement. The inset of Figure 6b shows the pattern of SERS responses when the target is illuminated by a wide aperture pump beam with λ = 0.532 μm (analyte concentration—10^−12^ M). The spectrum of one of the responses is shown in Figure 6b. As can be seen, it has a characteristic shape of the Raman spectrum of R6G molecules. The smallest detectable concentration of the analyte of 10^−13^ M is achieved with SERS mapping of the target surface, which, of course, is not a record result. Although this sensitivity is quite high and is achieved on a normal rough surface without taking special measures to increase the SERS response. Such measures could be the optimization of the thickness of the buffer coating, the production of an array of polygonal plasmonic nano-objects with sharp vertices and nanosized gaps between them, or in some other way, which we intend to implement in the future.

It should also be added that the experimentally achieved sensitivity of the SERS substrate with a size of 1.5 × 1.5 (mm), with a surface made using the same technology as the surface of the central target, was only 10^–10^ M, that is, it turned out to be three orders of magnitude worse than for the LITHS/S structure. This is another example illustrating the high efficiency of using the developed method of analyte enrichment in SERS-sensors.

## 4. Conclusions

This paper presents a fundamentally new technique that allows control of the evaporation of an uncharged liquid droplet on a hybrid superhydrophobic/superhydrophilic surface due to the action of a non-uniform electrostatic field. The analyte enrichment system, obtained by combining this approach with a special design for laser-textured hybrid superhydrophilic–superhydrophobic platforms, outperforms any other analyte enrichment system currently available and allows the realization of the targeted delivery of molecules of the substance to areas less than 0.0016 mm^2^. This result is more than six times less than that recently achieved of 0.01 mm^2^ deposition area. It potentially makes it possible to achieve a corresponding additional increase in the sensitivity of plasmonic sensors based on SERS, as well as to significantly increase the reliability and accuracy of the measurements at the atto-molar level. We believe that the approach presented is likely to be used for trace chemical detection in many areas.
